# Enhancing Sustainability and Resilience through Multi-Level Infrastructure Planning

**DOI:** 10.3390/ijerph17030962

**Published:** 2020-02-04

**Authors:** Jorge Salas, Víctor Yepes

**Affiliations:** 1 School of Civil Engineering, Universitat Politècnica de València, 46022 Valencia, Spain; jorsaher@doctor.upv.es; 2 ICITECH, Universitat Politècnica de València, 46022 Valencia, Spain

**Keywords:** multi-scale assessment, hierarchical relational modeling, cascading impacts, adaptive capacity, infrastructure integrated planning, road network, decentralization optimization

## Abstract

Resilient planning demands not only resilient actions, but also resilient implementation, which promotes adaptive capacity for the attainment of the planned objectives. This requires, in the case of multi-level infrastructure systems, the simultaneous pursuit of bottom-up infrastructure planning for the promotion of adaptive capacity, and of top-down approaches for the achievement of global objectives and the reduction of structural vulnerabilities and imbalances. Though several authors have pointed out the need to balance bottom-up flexibility with top-down hierarchical control for better plan implementation, very few methods have yet been developed with this aim, least of all with a multi-objective perspective. This work addressed this lack by including, for the first time, the mitigation of urban vulnerability, the improvement of road network condition, and the minimization of the economic cost as objectives in a resilient planning process in which both actions and their implementation are planned for a controlled, sustainable development. Building on Urban planning support system (UPSS), a previously developed planning tool, the improved planning support system affords a planning alternative over the Spanish road network, with the best multi-objective balance between optimization, risk, and opportunity. The planning process then formalizes local adaptive capacity as the capacity to vary the selected planning alternative within certain limits, and global risk control as the duties that should be achieved in exchange. Finally, by means of multi-objective optimization, the method reveals the multi-objective trade-offs between local opportunity, global risk, and rights and duties at local scale, thus providing deeper understanding for better informed decision-making.

## 1. Introduction

### 1.1. Implementation Planning as a Part of Resilient Planning

The concept of resilience was first introduced into ecological theory by Holling [1] as a measure of the capacity of a system to absorb change and external disturbance while maintaining key functions, and it is rapidly gaining ground in the urban sustainability literature [2]. In the field of urban infrastructure planning, resilient planning studies can refer to “planning” for a more resilient city, or to the “resiliency” of an urban planning, and, together, both approaches provide a constructive option for a controlled sustainable development of social–ecological systems [3]. While the first aspect focuses on the planning of actions leading to the improvement of a city’s resilience, the second has to do with the implementation of these actions within an urban framework. Resilient infrastructure planning, in this context, refers to a more flexible, adaptable approach for dealing with dynamic problems arising from the implementation of an infrastructure plan. This means that resilient infrastructure planning requires not only the design of measures for the improvement of infrastructure resiliency, but also measures ensuring the best implementation of these actions [3]. However, there are currently few methods incorporating the design of an implementation strategy as a part of the planning process across multi-level governmental environments [4,5].

### 1.2. Implementation Planning and Decentralization

Several authors have pointed out the role of decentralization in providing urban systems with their required adaptive capacity. Sharifi and Yamagata [6] pointed out that decentralization is essential for enhancing local adaptive capacity, and that a shift towards bottom-up planning approaches must be made in order to improve the adaptability and flexibility of urban systems, and therefore contribute to achieving sustainable urban development. Gonzales and Ajami [7] proposed a methodology for improving resilience by adding flexibility at a local scale in urban water systems, while Leigh and Lee [8] showed how decentralization leads to greater adaptability of water systems for specific local contexts and operational changes. Additionally, Rogers [9] demanded that national policies and actions should be framed to facilitate local adaptation.

While recognizing the importance of flexibility at the local scale, resilient planning argues for the need of a regional and national perspective [2,9,10,11]. In the planning of road networks, this integrated outlook makes it possible to pursue overall objectives such as overall condition improvement [12,13,14,15,16] or safety performance [17], as well to contribute to the mitigation of the system’s structural imbalances [18] and vulnerabilities [4,19]. Given the link between road networks and other essential facilities, such as hospital or schools, and their role in induced community vulnerability [20], reducing these networks’ vulnerabilities and structural imbalances should be a primary objective for infrastructure planning. These pursuits, however, can be jeopardized by ignoring the negative cascading, cross-scale effects that actions taken at the local scale can bring to bear on global objectives [9,21,22,23,24], such as coordination problems in decentralized systems [25,26]. In other words, while decentralization contributes to the adaptive capacity demanded at the local scale, it also poses risks [5] and barriers [27] to the achievement of objectives at larger scales, which demands a proper balance of decentralization [11] that facilitates integrated planning formulation and its implementation [28].

### 1.3. Decentralized Systems: Balancing Adaptive Capacity and Hierarchical Control

**Figure 1:**
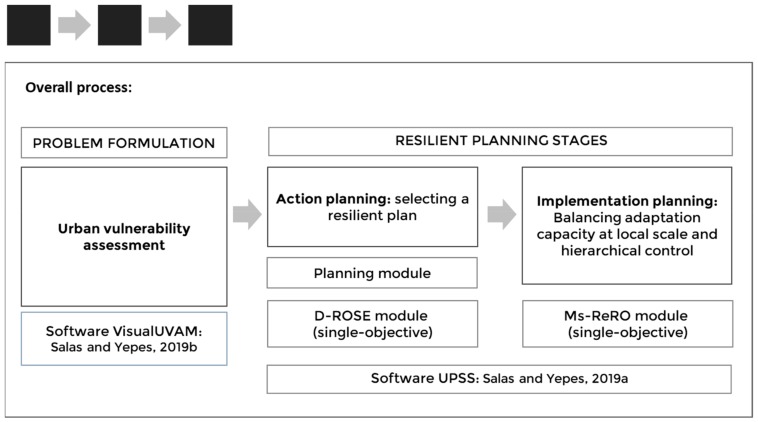
Overall process: vulnerability assessment and resilient planning.

The search for this balance between flexibility at the local scale and hierarchical control from central government has caused a debate among practitioners [27] whose ultimate purpose is to improve, in multi-level systems, the coordination between scales that is required [1,4,25,29,30]. This coordination, which is critical for implementing adaptation strategies in the transport sector [5] as well as in urban planning [27], highly depends on the system’s decentralization level [25,26]. Consequently, determining the proper decentralization level in multi-scale infrastructure networks is a key issue for a system’s design, implementation, and operation [2,31], and therefore for its resilient planning. However, there are currently very few studies affording implementation strategies that offer this balance between local adaptive capacity and the comprehensive perspective demanded for resilient planning. Ganzle et al. [32] pointed out the need for research specifically aimed at providing strategies for addressing the coordination problem arising from the implementation of integrated planning within multi-level governmental frameworks. Newman et al. [31] explored the effect of different decentralization levels in water systems, finding that a system’s performance may be sensitive to the level of decentralization adopted, while Roozbahani et al. [33] evaluated the risks of urban water supply systems from bottom to top by means of hierarchical structure analysis. Gupta et al. [34] pointed out the tension between top-down (centralized) and bottom-up (decentralized) planning approaches, and they stated the need to balance them for improved adaptive capacity. Regmi et al. [24] remarked on the convenience of integrating both planning approaches, the lack of methods addressing this objective, and the need to bridge the gap between global policies and local strategies. Finally, Salas and Yepes [4] presented Multi-scale relational risk and opportunity (Ms-ReRO), a methodology which respectively represents adaptive capacity at the local scale and hierarchical control at the global scale as “right” and “duty” rules between hierarchically linked entities [25,26]. This method combines optimization for the design of plans of action (Figure 1, planning module) with quantitative risk assessment and multi-objective optimization (Figure 1, Ms-ReRO module) in order to afford decentralization configurations to minimize overall risks and maximize local adaptive capacity. These decentralization configurations are defined via the “rights” and “duties” embodied in the relational contracts linking entities of interdependent scales [25]. Through these contracts, top entities (i.e., countries) transfer some of their “right” to take decisions to entities below (i.e., regions) which, in exchange, must achieve a given “duty”, or level of performance. By allowing these “rights” and “duties” to be regulated, the proposed framework enables the optimization algorithm to identify the trade-offs between risks and opportunities (Figure 1, Dynamic risk and opportunity simultaneous evaluation (D-ROSE) module), which makes it possible for the decision-maker to balance adaptive capacity and hierarchical control (Figure 1, Ms-ReRO module).

However, in their article, Salas and Yepes [4] studied risks only from the economic cost perspective, pointing out the need to introduce additional objectives in future work. They also remarked on the limitations of their methodology in providing criteria for choosing among pareto-optimal decentralization alternatives, which requires a deeper analysis of the trade-offs between rights, duties, and global and local risks and provides opportunities for enhanced decision-making.

The aim of this paper was to contribute to the field of resilient planning by enabling, for the first time, a multi-objective balance of local adaptive capacity and global risk control in net infrastructure planning, as well as to provide a deep analysis of the trade-offs between decentralization configurations and the risks and opportunities they bear for multiple objectives. By means of the proposed resilient planning process, both actions and their implementation were planned, in a decentralized system case study, in order to mitigate the system’s urban vulnerability, to improve the road network’s current condition, and to minimize the economic cost.

The remainder of this paper is organized as follows. In the Methods section, each stage of the three-step process (Figure 1), namely urban vulnerability assessment, action planning, and implementation planning, is described. In the Case Study section, the whole process is illustrated through an actual case, the results of which are presented in the Results section. These results are then analyzed in the Discussion section to show whether the applied method contributed to resilient planning or not, and, finally, general conclusions are drawn in the closing section.

## 2. Methods

### 2.1. Step 1: Urban Vulnerability Assessment

Broadly, vulnerability can be understood as the susceptibility to suffer from, or the difficulty in coping with the negative effects of an event, and it has become a major concern for sustainable urban development [35,36,37,38]. In a prior work, Salas and Yepes [39] presented VisualUVAM, a software that affords the urban vulnerability assessment of cities, provinces, and regions of Spain. This software extended the scope of possible variables for the characterization of urban vulnerability (UV) from the three basic criteria adopted by the Spanish Observatory of Urban Vulnerability (OVU) to a wider set of 36 possible indicators, among which the method selected those most suitable according to several criteria. Based on this set of the most suitable indicators, the method yields a quantitative assessment of both the state of vulnerability at the end of a given time period and the risk of becoming more vulnerable during the next period.

**Figure 2:**
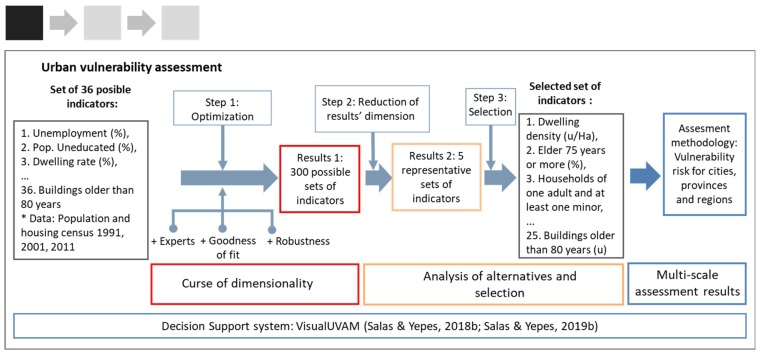
Step 1, urban vulnerability assessment: selection of indicators.

In VisualUVAM, the selection among the 36 possible indicators for characterizing UV is addressed via a multi-objective optimization (MOO) problem in which expert judgment, statistical consistency, and robustness against data uncertainties are used as the criteria for the choosing of indicators (Figure 2).

Since MOO usually yields large sets of solutions, giving rise to the so called “curse of dimensionality” problem [40], the assessment process also implements a cluster-analysis-based methodology that synthesizes the initial space of 300 solutions into a smaller, manageable one of 5 representative solutions (Figure 2) [39]. This enables the decision-maker to focus the analysis on the most promising alternatives and to select the most suitable, which affords a multi-scale evaluation of the risk of urban vulnerability of entities at city, province, region, and country scales [39]. Once the set of indicators has been selected, the method yields, for each of the cities, provinces, and regions being assessed, both the state of vulnerability (SV) at a given time and the risk of increasing vulnerability in the future.

### 2.2. Step 2: Resilient Planning I—Action Planning

The Urban Planning Support System (UPSS) [4] is a piece of software, programmed in Matlab, affording both the action planning and the implementation planning demanded by resilient planning. This software, however, still suffers from the lack of multi-objective capacity that this paper attempted to overcome. As to the action planning, UPSS includes planning and D-ROSE modules for the generation of planning alternatives and for evaluating the alternatives’ risks and opportunities, which enables an informed selection of the most adequate planning alternative.

#### 2.2.1. Planning Module: Generation of Planning Alternatives

**Figure 3:**
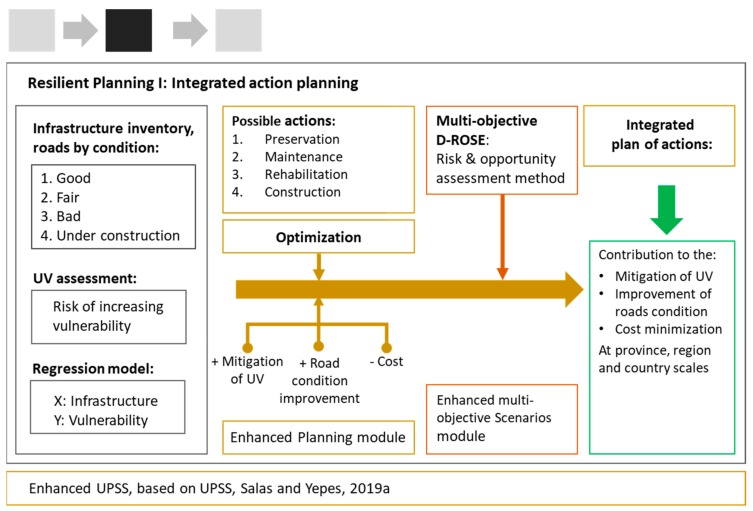
Step 2: planning of actions and action risk analysis.

Based on the infrastructure inventory (Figure 3), the planning module sought that combination of possible maintenance and construction actions [41] that would maximize the performance of the investment strategy according to three objectives, namely the mitigation of urban vulnerability, road condition improvement, and economic cost.

Objective 1 was the mitigation of urban vulnerability. To evaluate the contribution of infrastructure to urban vulnerability mitigation, we first built a regression model based on the results of Step 1 and the road network’s condition as described by the infrastructure inventory [4], which estimated the evolution of the risk of urban vulnerability in terms of the evolution of the road network’s condition (Figure 3). This allowed the formulation of the urban vulnerability mitigation objective as follows:

UVMNet = ∑i,jUVM(RCV(Planj, Invj), Modi)(1)

where UVMNet is the urban vulnerability mitigation impact of the road network, i is each of the infrastructure system’s hierarchical scales, j is each of the entities in the i scale, k is each of the actions planned for the j entity, and UVM is the evaluation of the RCV(Planj, Invj) road condition variable’s evolution of the entity under the Modi regression model.

Objective 2 was condition improvement. Building on prior work [4], we linked possible actions with condition improvement, which enabled us to estimate the condition improvement that a given set of actions would produce on the infrastructure inventory at the end of the analyzed period (Table 1). As we were planning for a 10 year period, in the case of actions with a shorter service life increase (Table 1, SLI) we assumed their repetition until the completion of the planning period [15]. For example, in the case of preservation, a treatment with a service life of 2.5 years, this action was considered to be applied four times over the 10 years of the analysis period (Table 1, column “Treatment/Period”).

Finally, we formulated the road network’s condition improvement objective as the sum of the pavement condition index condition score (PCI-CS) improvements of all the entities of the road network being analyzed (Matin et al., 2017 [12]):

RCI_Net_ = (∑_j_ ΔRC (Plan_j_, Inv_j_) × CS)/∑_j_ R (Plan_j_, Inv_j_) (2)

where RCINet is the road network’s condition improvement of the j entities of the network, ΔRC is the transference function that transforms, based on Table 1, the actions of the Planj carried out over its Inv_j_ inventory into the evolution of the road condition variables, PCI-CS is the condition score attached to the road condition variables (Table 2), and R(Plan_j_, Inv_j_) is the quantity of roads in all conditions after carrying out the infrastructure plan.

Objective 3 was economic cost. As to the economic cost objective, the cost of each road network planning alternative was formulated as the product of the actions included and their unitary costs:

EC_Net_ = ∑I_i,j,k_ Action_(i,j,k)_ × ICost_(i,j,k)_ × IcostAsymm_(i,j)_ (3)

where ECNet is the plan’s cost, and Action(_I,j,k_) and ICost(_I,j,k_) are, respectively, the quantification of the actions included in the plan and the unitary costs of each of the k planned actions. IcostAsymm is a normalized asymmetry index that reflects different investment costs by entities of a given context, e.g., counties of a given province, provinces of a given region, or regions of a given country [4].

Objective 4 was the performance of the most vulnerable entities group of interest. Finally, in order to incorporate equity into the planning process and to provide proper visibility to the most vulnerable [6,42], we introduced as an additional objective the ratio between the most vulnerable group’s performance [4] and the overall performance in the “Condition improvement” objective:

RCI_Vul_ = RCI_Net/_RCI_Hv_ (4)

where RCIVul is the road condition improvement ratio of the most vulnerable entities, while RCINet and RCIHv are, respectively, the net and the highly vulnerable entities group’s condition improvement scores.

#### 2.2.2. Scenario Module: Evaluation of Risk and Opportunities

The planning process implemented D-ROSE (Figure 3), an uncertainty analysis method capable of identifying a set of relevant scenarios and evaluating the risks and opportunities that these scenarios entail for each of the possible planning alternatives [4]. This method, however, lacks the multi-objective capacity required for analyzing planning alternatives against multiple risks [4], as was the case here. This multi-objective capacity implies that, for a proper selection of the most adequate planning alternative, the decision-maker should be enabled to simultaneously visualize the risks and opportunities borne by the set of relevant scenarios from all points of view, i.e., regarding all objectives. To address this, interactive visual analytics use different data visualization techniques, offering multiple, linked views of relevant information. Therefore, we implemented in the planning tool the capacity to simultaneously display risks and opportunities for all the objectives and planning alternatives to understand the trade-offs between the different risks, opportunities, and possible decisions [43].

### 2.3. Step 3: Resilient Planning II—Implementation Planning

**Figure 4:**
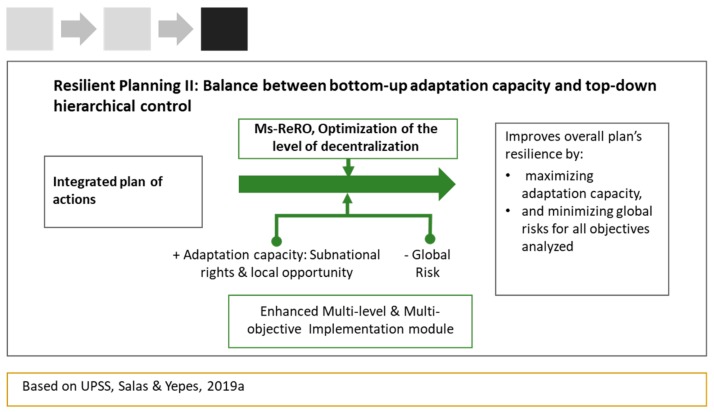
Step 3: implementation planning and risk analysis.

As a final step, the process of resilient planning required the design of an implementation mechanism [44] that affords a proper balance between hierarchical control and adaptive capacity at the local scale across the road network’s decentralization structure (Figure 4).

Ms-ReRO [4] is an uncertainty analysis method specifically designed for this purpose, based on hierarchical probabilistic relational modeling (HPRM) [45] and MOO, which affords an assessment of the global risks and opportunities at the central government (top) scale triggered by a plan’s implementation at the municipal (local) scale. In this methodology, integrated planning implementation is represented as a hierarchical system of systems that are connected by relational contracts, and risks and opportunities are derived as the bottom-up cascading impacts produced by the actions performed at a local scale. In decentralized infrastructure systems, contracts between parties are key elements in the implementation scheme [23,25]. Contractual arrangements prescribing very precise actions work well at a tactical scale but not at a strategic, long-term scale [23]. Instead, Ms-ReRO includes a more flexible contractual framework based on the concept of relational contract [25], which defines “right” and “duty” [25,26] rules across contracting parties. By means of this, the proposed framework allows top entities to transfer the “right” to vary the initial plan to the entity below, which, in exchange, is obliged to achieve a given outcome, i.e., to perform a “duty”.

In HPRM, actions are performed at the bottom scale and their consequences are then bottom-up propagated, therefore impacting at the top scale; the aim is to regulate these actions and impacts by means of the relational contract’s rules. In consequence, we first allowed variation the quantities specified in the baseline plan within certain limits (rights), which were modeled as the lower and the higher bounds for each action. We then imposed, as a restriction of the choice between the right’s bounds for each action, that their joint effect had to fall within a given performance range, which we called “duty” and which represented the maximum possible deviation from the performance expected to be accomplished by each entity in the integrated plan. In consequence, in terms of the generation of simulations at the bottom scale, which will subsequently be bottom-up propagated through the relational system, we only admitted those meeting the conditions specified by the relational contracts at the bottom scale, i.e., we eliminated failing simulations from the set of possible realizations generated by the Monte Carlo simulation method. Finally, the choosing of actions between the rights’ bounds by local entities is affected by their behavioral preferences, which we formalized by means of triangular probability distribution functions (PDF) functions. For this purpose, we employed a stochastic approach since it allowed integration into a single object of the rights’ upper and lower bounds and the local preferences as the lower, central, and upper points of a triangular PDF function [4].

This relational framework enabled, through MOO, the balancing of hierarchical control and adaptive capacity by simultaneously minimizing the risk of failure at the top scale (global) while maximizing the opportunity to achieve better performance at the local scale.

Decentralization Objective 1 was global risk minimization:

RGlobal(AP,IP) = P(FAP,IP,T) × I(FAP,IP,T)(5)

where RGlobal (AP,IP) is the risk, for the implementation plan IP of the action plan AP, of achieving a result worse than that of the failure condition F; P(FAP,IP,T) is the probability of achieving anF failure condition at the system’s T top scale; and I(FAP,IP,T) is the impact of this failure. In Ms-ReRO, the F failure condition consists of a performance worse than the previously set up pessimistic threshold.

The probability of failure, in turn, was defined as

P(F_AP,IP,T_) = N(Sims^F^_AP,IP,T_)/N(Sims_AP,IP,T_) (6)

where N(Sims^F^_AP,IP,T_) is the number of simulations achieving failure, while N(Sims_AP,IP,T_) is the total number of simulations performed following the method described by Salas and Yepes (2019a).

Finally, failure’s impact was formulated as

I(F_AP,IP_) = mean(f(Sims^F^_AP,IP_)) − f(BL_AP,IP_) (7)

where mean(f(Sims^F^_AP,IP_)) is the mean of the performances achieved by failing simulations, and f(BL_AP,IP_) is the value achieved in the realization of the baseline plan of actions.

Decentralization Objective 2 was local opportunity maximization. Conversely to the risk, we modeled opportunity based on the simulations improving a given level of performance. Therefore, opportunity at local scale was formulated as

O_Local_(AP,IP) = P(W_AP,IP,B_) × I(W_AP,IP,B_) (8)

where P(_WAP,IP,B_) is the probability of achieving a “W” windfall condition at the system’s “B” bottom scale, I(_WAP,IP,B_) is the impact of the windfall condition, and B is each of the entities at bottom scale.

Decentralization Objectives 3 and 4 were related to the relational framework’s flexibility maximization. As to the improvement of entities’ capacity of varying the plan, we implemented this by maximizing the sum of the means of the rights bestowed by scale across the whole relational system. This flexibility was also improved via maximization of the range within which each entity was allowed to deviate from their duties, i.e., from their intended result.

In sum, decentralization Objectives 2–4 represented the maximization of the system’s adaptive capacity at local scale, while decentralization Objective 1 accounted for the minimization of the system’s risk of failing in the attainment of the required global performance. In seeking to achieve these goals, the MOO problem operated over the “rights” and “duties”, which therefore became the MOO’s decision variables, and were formulated as the percentage in which entities are allowed to deviate from the baseline plan, in the case of rights, or from the expected performance in the case of duties [4].

## 3. Case Study: Resilient Road Network Planning in Provinces of Spain

### 3.1. Information Collection Process

#### 3.1.1. Information Required for Urban Vulnerability Assessment

Following prior work, the compilation of the quantitative information was downloaded from the website of the National Institute of Statistics, comprising 36 indicators for each of the 403 cities (264 of which are from the province of Valencia), 52 provinces (including Ceuta and Melilla), and 19 regions (including the autonomous cities of Ceuta and Melilla as regions) that composed the elaborated database [39]. This information was collected for the years 1991, 2001, and 2011, allowing analysis of the evolution of urban vulnerability in the periods 1991–2001 and 2001–2011.

Along with the quantitative information, we also gathered the qualitative information regarding experts’ preferences for the indicators best representing urban vulnerability, required by the assessment process [39]. Based on the analytic hierarchy process (AHP) multi-criteria technique [46], we asked the experts to pairwise compare the 36 indicators of the quantitative database, which were structured in three levels so that only in one case was the number of indicators to be compared greater than five. Basically, this structuring of indicators was a transposition of the conceptual framework adopted by the Spanish OUV, to which some indicators were added.

Further, to avoid the problem of inconsistent judgment elicitation [47], we developed a software application, programmed in Matlab, that provided experts with real-time feedback on their judgments’ consistency, enabling them to interactively revise their judgements until they became acceptable [39]. As an outcome, we obtained the experts’ relative preferences for indicators as weights, which were incorporated into the experts’ preferences objective in the optimization process (Figure 2).

#### 3.1.2. Information Required for Urban Infrastructure Planning

As to the gathering of quantitative information on road conditions, we resorted to the data available from the Local Infrastructure and Equipment Survey (EIEL), which included a wide range of infrastructures present in municipalities of 50,000 habitants or fewer in all Spanish regions, with the exception, due to their specific organizational regimes, of the Basque Country and Navarra [4].

Since the planning process required a regression model correlating the evolution of urban vulnerability and that of the condition of urban infrastructure (Section 2.2.1), we retrieved from the EIEL the data corresponding to those employed for the assessment of UV in Step 1 (Section 2.1), i.e., between the years 2000 and 2010, and structured it based on the city, province, and region (autonomous communities) scales. However, since in Spain, road network planning is under the jurisdiction of the state, regions, and provinces, but not of cities, we excluded the latter scale from our database and settled on provinces as the bottom scale. We then sought to achieve objectives at the national (top) scale by building planning alternatives from a provincial scale, which is an approach more akin to actual road network decision-making than doing it from a municipal perspective.

### 3.2. Running of the Process

#### 3.2.1. Step 1: Urban Vulnerability Assessment

The assessment of urban vulnerability was performed via VisualUVAM, a software that covered all the steps of the urban vulnerability assessment process described in the methodology (Section 2.1). Following the guidance afforded by the software, we first generated a set of 300 pareto-optimal combinations of indicators which, by means of the visual analytics and cluster analysis techniques implemented in the tool, were synthesized into a more manageable set of nine possible combinations. We then undertook a process of analysis that culminated in the selection of the combination of indicators deemed most appropriate [39].

#### 3.2.2. Step 2: Action Planning

Based on the results of the urban vulnerability assessment carried out in the previous step, and on the gathered information of the road network’s condition, the UPSS planning module (Section 2.2.1) provided an initial set of 300 pareto-optimal planning alternatives (Figure 3). The planning alternatives were then filtered by means of the implemented cluster analysis method [39], reducing the initial set of 300 possible solutions to a set of 11 representative, relevant alternatives, which were further analyzed by the scenario module (Section 2.2.2). By means of D-ROSE, we generated random scenarios and evaluated the risks and opportunities that these scenarios bore for each relevant planning alternative. In this case, we employed the scenario module to swap the range of possible decentralization combinations and therefore represent the impacts that different levels of decentralization had on each possible plan. Subsequently, trade-offs between risks, opportunities, and planning alternatives were evaluated and, after the analysis of these results, the most adequate plan was chosen for implementation.

#### 3.2.3. Step 3: Implementation Planning

Based on the planning alternative selected in Step 2, the UPSS implementation module (Section 2.3) simultaneously sought, through the optimization of the system’s level of decentralization, the minimization of global risks and the maximization of local adaptation (Figure 4). This afforded a set of optimal configurations of the relational contract’s rights and opportunities, from which it was possible to draw out the trade-offs between global risk and local adaptive capacity for each objective. These trade-offs were then analyzed from a multi-objective perspective, which enabled us to balance different risks, opportunities, and possible decisions and accordingly choose the most adequate implementation plan.

## 4. Results

### 4.1. Step 1: Urban Vulnerability Assessment

**Figure 5:**
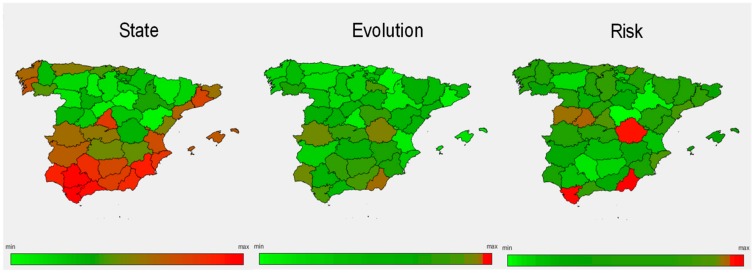
Results by province of the urban vulnerability assessment process: UV State in 2011, evolution of UV state between 2001 and 2011, and risk of increasing UV from 2011 onwards.

Figure 5 shows the results of the state of UV, the evolution of UV state, and the risk of increasing UV for provinces of Spain, which revealed how urban vulnerability is, in general, more present in coastal and highly populated provinces [39]. Based on this information, the 30% most vulnerable entities were identified and grouped for the incorporation of their specific interest in the search, in Step 2, for optimal infrastructure plans.

### 4.2. Step 2: Planning of Actions

**Figure 6:**
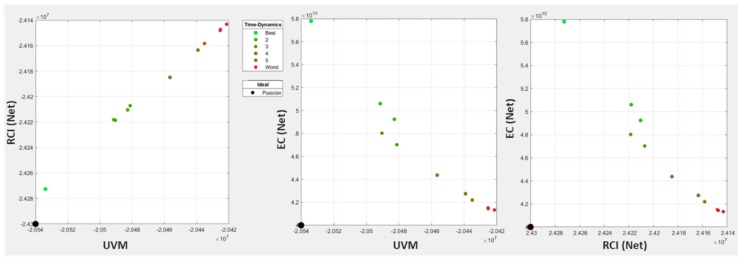
Trade-offs between objectives.

The trade-offs between planning objectives (Figure 6) showed that the overall urban vulnerability mitigation (UVM (Net)) and road network condition improvement (RCI (Net)) objectives were aligned, which was consistent with the idea of the contribution of net infrastructures to the mitigation of urban vulnerability [4,19]. These objectives were also aligned with the maximization of the RCI(Vuln), which expressed the ratio of road condition improvement of the most vulnerable entities within the total, showing how, in some cases, it was possible to reconcile particular interests with general interests. As expected, all these objectives were in conflict with the network economic cost (EC (Net)) minimization which, since the results were pareto-optimal, could be used as an ex-post budgetary restriction by setting up the maximum economic cost allowed in the implemented selection controls.

**Figure 7:**
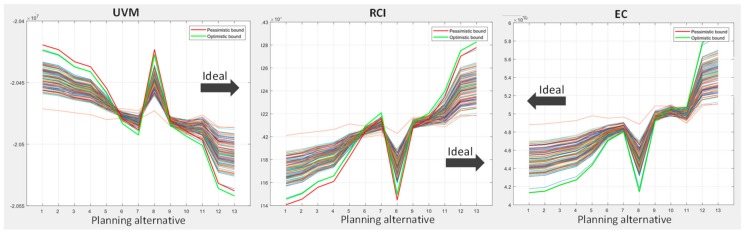
Results of the Monte Carlo simulation for each planning alternative. Simulation results exceeding pessimistic or optimistic bounds bear, respectively, risks or opportunities. The order of the planning alternatives in the horizontal axis indicates lower to higher UVM performance.

Figure 7 presents, for each objective, the results of the Monte Carlo simulation carried out over each of the planning alternatives, and also reflects the direct correlation between closeness to ideal and worst risk and opportunity performance. Relevant solutions performing well at the UVM objective did so at the RCI, while they performed badly at the EC objective. In effect, as we moved toward the right (ideal) in planning alternatives for the UVM and RCI objectives, the simulations’ results passed from above the optimistic threshold to below the pessimistic threshold, which indicated movement from opportunity to risk. Conversely, at the EC objective, which was opposite to UVM and RCI, better (cheaper) solutions were placed at the left and worse (expensive) at the right, and, consequently, simulations improving the expected performance are on the right, while those worsening it are on the left side.

**Figure 8:**
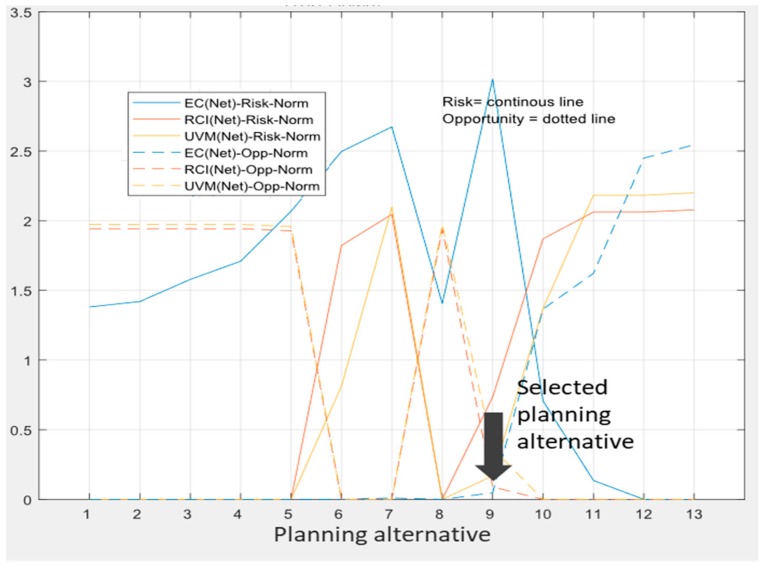
Normalized risks (Risk-Norm) and opportunities (Opp-Norm) of planning alternatives by objectives in terms of distance, in standard deviations, from the lowest risk/opportunity.

As to the multi-objective analysis of the results, Figure 8 portrays the risks and opportunities of each planning alternatives for the set of 100 scenarios generated. The analysis of these results showed that planning alternatives 7, 8, and 9 were the most relevant for our decision, since in all objectives they represented the turning point from opportunity to risk or vice versa. Finally, we selected alternative 9 due to our bias toward solutions improving especially the condition index of the most vulnerable entities, which in Figure 8 were to the right.

Each of the generated planning alternatives is a baseline plan specifying the quantity of each possible action that should be carried out at the bottom (provincial) scale to bring about the planned performance at the top (country) scale. Table 2 shows the specific results of the selected plan for the region of Comunidad Valenciana and its provinces.

### 4.3. Step 3 Implementation Planning

**Figure 9:**
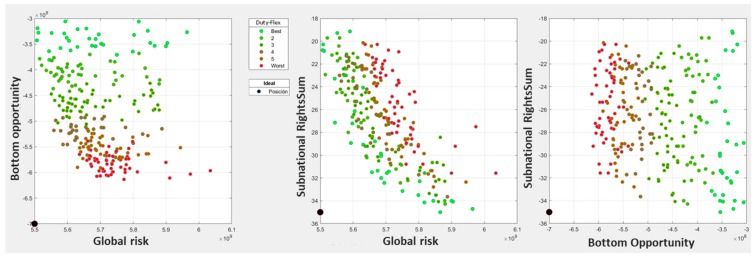
Results of the generation of decentralization solutions, trade-offs between global risk, opportunity at local scale, summation of sub-national rights, and flexibility in duties for the economic cost objective.

The implementation planning module (Section 3.2.3) afforded a set of pareto-optimal solutions for the configuration of the rights and duties which made up the system of relational contracts. As shown in Figure 9, economic risk reduction at the top scale was inversely correlated with opportunity increase at the local scale, which, on the other hand, had a clear inverse correlation with increase in the relaxing of duties. Finally, Figure 9 also shows a strong inverse correlation between increasing rights at subnational scales and reducing economic risks at the national scale, which, on the other hand, was directly correlated with increasing flexibility in duties. Altogether, the set of solutions showed that increasing local opportunity was in opposition to global risk reduction, and that increasing rights led to increased global economic risks but not to increased economic opportunity, thus producing an asymmetry in the share of risks and opportunities. By increasing rights, we increase global risk, but we do not necessarily increase local opportunity. As to the relaxing of duties at the bottom scale, its increase was slightly associated with reductions of both risks and opportunities. However, when this increase came together with that of the rights, it played against risk reduction at the top (national) scale (Figure 9).

For a better understanding of how the decentralization model works, we resorted to global sensitivity analysis to evaluate the decision model in terms of output uncertainty and factor importance in order to gain a better understanding of how the model parameters affected the final outputs [48]. Regression-based and variance-based methods are two of the most commonly used approaches for global sensitivity analysis, and they perform almost equally well for quantifying output variance and contribution to variance of the input parameters, especially in the case of relatively small input uncertainties [49]. By incorporating the Matlab code [50] developed by Groen et al., [49] into our own Matlab software, we performed a global sensitivity analysis based on the squared standardized regression coefficients method. The results of the global sensitivity analysis showed that rights and duties at the province (bottom) scale were the driving factors in all objectives, but they were unequally distributed along objectives (Table 3). While duties at the province scale was the factor with the highest impact on global risk in the economic cost and mitigation of UV objectives, it had little impact on the road condition improvement global objective. Conversely, rights at the province (bottom) scale was the driving factor for opportunity at the bottom scale for all objectives, but also posed global risks for the road condition improvement objective.

**Figure 10:**
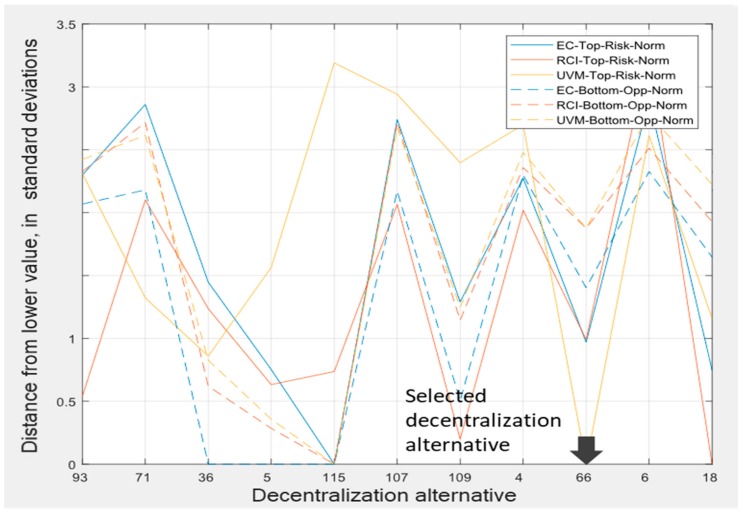
Implementation risks and opportunities borne by the representative decentralization solutions.

As to the selection of the proper decentralization configuration of the relational model, we resorted to cluster analysis to synthesis the initial set of solutions into another more manageable set, which we analyzed from the perspectives of all the objectives involved in the planning process (Figure 10).

Decentralization alternatives 115, 66, and 18 achieved the lowest risks in terms of the economic cost, urban vulnerability mitigation, and road condition improvement, respectively, showing that there was not a unique best solution for all objectives and that some qualitative analysis is required to perform such selections. Alternative 115 also had the fewest opportunities of all, while alternatives 66 and 18 were the only alternatives with greater opportunity than risks in all objectives. Of these, alternative 18 presented a slightly better balance between risk and opportunity.

On the other hand, the performed global uncertainty analysis allowed some management implications to be drawn out [51]. On one hand, the focus should be put on the rights and duties bestowed at the bottom rather than at the top scale. On the other hand, there is no formula for balancing rights and duties that can be indiscriminately applied to all objectives, since relaxing duties, for example, would be risky at the global scale in terms of the economic cost and the mitigation of urban vulnerability points of view, but not much from a road condition improvement outlook, which would be highly affected, instead, by increased rights. Therefore, it is necessary to balance not only risk and opportunity, but also the objectives, selecting planning alternatives with better balance in those objectives prioritized by the decision-maker. In consequence, despite alternative 18 having an overall risk and opportunity balance slightly better than alternative 66, we selected the latter due to its lowest risk in the urban vulnerability mitigation objective, which we prioritized over the road network condition improvement. We therefore selected alternative 66, which enabled us to set up the relational contracts required for the implementation of the planning alternative (Table 4).

## 5. Discussion

### 5.1. Action Planning

The MOO approach yielded trade-offs between the objectives involved in the planning process, i.e., maximization of urban vulnerability mitigation and road network condition improvement and minimization of economic cost. Additionally, it provided valuable information on the specific effects of the planning alternatives over the most vulnerable entities which, together with the analysis of the results of the risk and opportunity assessment, enabled us to select the most suitable planning alternative for its further implementation.

From a strategic point of view, each planning alternative represented a baseline plan containing the basic determinations required for the road network’s maintenance and construction integrated planning, comprising regions and provinces of Spain. The solutions provided, based on the road network’s current condition, the quantity of each action that should be performed for each entity at the bottom scale to attain a given performance at global scale, enabling their further development at the tactical level via relational contracts.

### 5.2. Implementation Planning

The results yielded by Step 3 showed how different decentralization solutions led to different risks and opportunities in the implementation of the selected planning alternative, and how the proposed methodology can be employed to find the most convenient decentralization solution. By means of this, the method afforded proper balance between risks at the top (national) level, opportunities at the bottom (provincial) scale, and rights bestowed through relational contracts. These rights represent the capacity to select actions other than those of the baseline plan, and, in consequence, are a way in which local entities can adapt the integrated planning to their circumstances and specific needs. Opportunity at the local scale, on the other hand, represents the potential positive effect that rights might have on local entities’ performance, which is strongly correlated with subnational rights. Together, rights and local opportunity account for the demanded planning system’s local adaptive capacity [7,9]. This plan’s flexibility, as shown in Section 4.3, was in conflict with the reduction of risks at top scale, which reinforced the idea that, in infrastructure hierarchical systems, resilience at local scale does not necessarily lead to resilience at the global scale [9,23], and that some balance between global objectives and local adaptation is required [11,28,29]. Ms-ReRO addresses this issue by means of multi-objective optimization, which in our case afforded a set of optimal decentralization solutions from which it was possible to select an implementation plan achieving the demanded balance between global risk and local adaptive capacity.

Based on the trade-off between top-risk minimization and local adaptive capacity maximization, it was possible to select the proper action implementation plan, which included the guidelines required for issuing a system of relational contracts (Table 4). Contracts play a key role regarding the level of resilience level in a fragmented or decentralized infrastructure system [23], and should afford the means for dealing with the uncertainty always present in any infrastructure system’s integrated plan’s implementation and operation [25]. Relational contracts are a kind of contract specifically designed to alleviate relational problems between hierarchically dependent entities of decentralized systems [25], allowing the incorporation of both rights and duties, and they therefore provide the best framework for materializing the method’s results. This approach, also allows multiple objectives to be taken into account by specifying in the relational contracts multiple duties to be carried out, which in our case were the expected performance of each entity in urban vulnerability mitigation, road network condition improvement, and economic cost.

**Figure 11:**
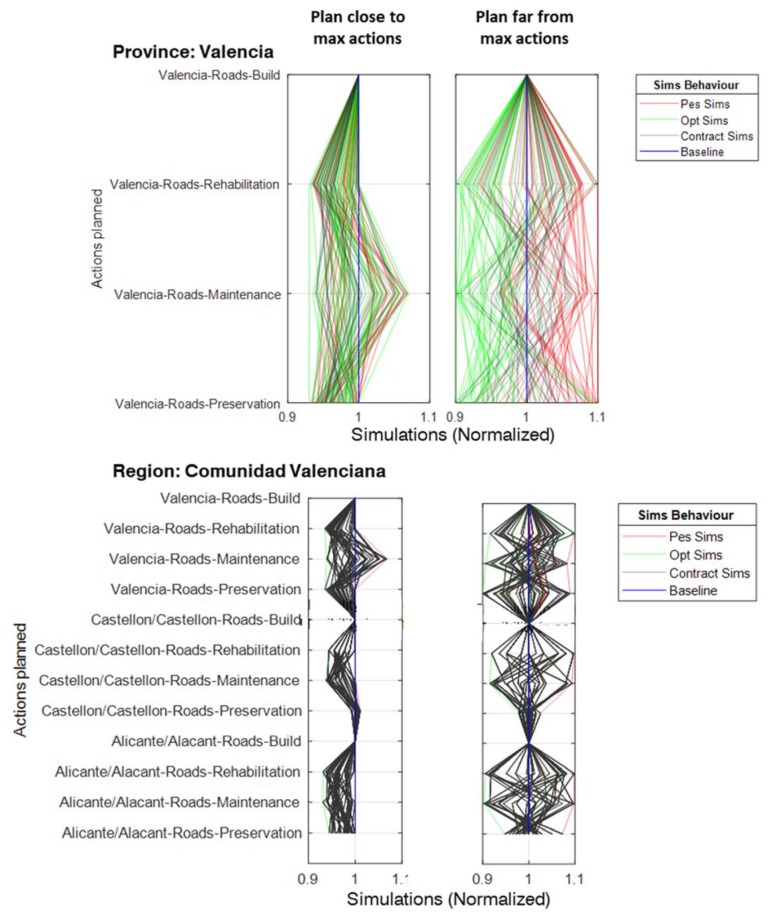
Example of simulations generated at provincial (bottom) and regional scales for different planning alternatives. Contractual simulations at the bottom scale were propagated to top scales. The decentralization configuration in both cases was the same.

As to the relationship between planning alternatives and implementation risks and opportunities, the results showed that, for each objective, planning solutions close to the ideal were prone to risk. In our case, this was due to the fact that in planning alternatives already close to the maximum or minimum possible quantity of a given action, transferring rights beyond this limit will be ineffective, thus producing an asymmetry in the PDF describing each entity’s possible actions. For example, in a planning alternative preserving 97% of the roads in a good state, i.e., close to the maximum possible preservation quantity, bestowing rights of 15% means that the theoretical upper bound will exceed the real one, rendering ineffective 80% of the theoretical potential increase of actions. On the other hand, for the same example, its lower bound will fall from 95% down to 82.45%, thus producing an asymmetry that will be reflected in the behavior of the simulations generated (Figure 11), and therefore in the risks and opportunities attached to this decentralization configuration. This phenomenon has important implications for the issue of relational contracts, since their actions’ upper and lower bounds will not necessarily match the range expressed by the rights embodied in the contract. In consequence, it is necessary to explicitly define, for every relational contract, the rights as the action’s lower and upper bounds instead of only as a range (Table 4).

As to how the plan’s adaptive capacity, which is an abstract concept, can be materialized by local entities, Figure 11 plots, labeled as “contract sims”, examples of possible variations over the baseline plan that could be carried out by local entities (provinces) without violating the relational contract, i.e., fulfilling the assigned “duties”. These variations at the local scale will then combine with those of other provinces of the same region to determine the joint effect on the region’s duties and so on, thus enabling the evaluation of the cascading impact of variations at a local scale over the objectives at the global scale. However, the presence of multiple duties–objectives embodied in the relational contract rules requires an additional control mechanism to simultaneously achieve them. In effect, this multi-objective dimension in the system of relational contracts means that variations at a local scale being acceptable from an economic point of view would not be acceptable from a road condition improvement outlook (planning alternative 114, Figure 10), and would therefore be rejected. Since there is not any one best solution for all objectives, a second-order analysis balancing not only risks and opportunities, but also the objectives themselves is necessary. In our case, we prioritized the minimization of risk for urban vulnerability mitigation and the balance between risk and opportunity, and therefore selected alternative 66; had we preferred risk minimization for road condition improvement, the best alternative would have been alternative 18.

The system of relational contracts helps in dealing with some kinds of uncertainty arising from the implementation of integrated plans across a territory, such as uncertainties related to the financial capacity of entities along the planning period. The proposed contractual framework enables local entities to adapt the baseline plan to their specific financial contexts by, for example, moving quantities from actions demanding heavy initial outlays, such as rehabilitation, to those requiring payments distributed over time, such as preservation. In contrast, it would be possible at some point that local entities have enough financial resources to undertake more demanding actions and are therefore willing to move quantities from preservation to rehabilitation, which they can do without trespassing upon the economic duty for the whole period. Another source of uncertainty would be the entities’ capacity to bring about the baseline plan, which may contain actions that are difficult for them to perform due to, for example, human resource limitations. In this case, entities can ask the upper scale to partially assume the implementation of the baseline plan or to adapt it by increasing those activities for which they have enough resources. Local entities may also have a better knowledge on which roads have strategic importance for them that, within the system of relational contracts, can be used to improve the baseline plan. In the hypothetical case of a province with roads in a good state that are not completely preserved but are more important than any of the roads in bad states that are planned to be rehabilitated, local entities can automatically move economic resources from rehabilitation to preservation according to their aim, provided they still achieve their duties.

Finally, in governmental contexts, there are always institutional disputes surrounding any integrated, long-term planning that can prevent its implementation. By changing the triangular PDF modeling the behavior of the actions affected by the dispute [52], the method allows assessment of the impact on the local and the global objectives resulting from this change, which could be of help in promoting agreement between parties.

## 6. Conclusions

Resilient planning demands not only resilient actions but also resilient implementation [53]. Despite the vast amount of research devoted to developing methods for the planning of resilient actions, there have been very few studies investigating plans’ implementation [4], which, in the case of net infrastructure planning, requires a proper balance between global risk minimization and local adaptive capacity maximization [9,11,28,29]. This paper contributes to resilient planning by, on one hand, extending the initial capacities of UPSS [4] to the search for road network investment plans and decentralization alternatives that are optimal from the perspectives of the network’s condition improvement [54], contribution to urban vulnerability mitigation, and minimization of the economic cost. By integrating social sustainability aspects as a relevant criterion for the decision-making process, the method facilitates the adoption of a resilient plan of action, contributing to more sustainable development. On the other hand, this paper provides planners with a novel way of materializing a plan’s adaptive capacity at the local (bottom) scale and risk control at the global (top) scale. By means of the rights and duties included in the provided decentralization solution, it is possible to set up a system of relational contracts in which the integrated plan is transferred from national to provincial entities, where it is finally executed according to the relational contract specifications.

Along the process, the improved planning support system afforded a plan of action for the Spanish road network with the best balance between closeness to ideal and risks entailed from a multi-objective perspective (Section 4.2). Additionally, the planning process provided a decentralization solution for the best implementation of the plan of actions across the Spanish governmental structure, consisting of the country, regional, and provincial levels (Section 4.3). This decentralization solution was then used in a novel way to shape a system of relational contracts between hierarchically dependent entities in which local adaptive capacity was formalized as the right to vary, within certain limits, the plan of action being implemented, and global risk control was materialized by means of the duties that should be achieved in exchange of the rights conferred. Overall, the presented method integrates, for the first time, the planning of resilient actions with the planning of their resilient implementation from a multi-objective point of view, thus contributing to the field of resilient planning.

In the selection of the most adequate planning alternative, the multi-objective capacity allowed the identification of key planning alternatives from the risk and opportunity points of view and, based on the alternatives’ impact on the most vulnerable entities, the selection of the most appropriate one, contributing to the incorporation of equity into the planning process [42]. The results showed, on the other hand, that there was a clear relationship between closeness of the planning alternatives to the ideal and increased risks and, vice versa, alternatives farther from the ideal point were prone to opportunity, i.e., they had more chances of improving their expected performance. Regarding the selection of the proper implementation plan, the method’s multi-objective capacity revealed that there were no clear trade-offs between the objectives’ global risks and local opportunities. Instead, it was necessary to separately evaluate decentralization alternatives and select the most adequate according to the balance between risks and opportunities and the decision-maker’s preferences for objectives. This evaluation prevents alternatives being chosen that perform well in a less important objective and badly in those more relevant to the decision-maker, as it affords improved global risk control in which adaptive capacity at the local scale is bound to the simultaneous accomplishment of a given level of performance for each objective. This paper also presents a novel approach for materializing a plan’s adaptive capacity into actions. The use of relational contracts allows the contractual formulation of adaptive capacity as the rights bestowed to local entities, which enables them to vary the baseline plan to adapt it to their local circumstances and needs, in exchange for carrying out the duties assigned.

Additionally, the paper provides valuable insights into the relationships between planning objectives, planning alternatives, and their implementation’s global risks and local adaptive capacity. As to the planning objectives, the results showed that the mitigation of urban vulnerability and road condition were aligned objectives, which was consistent with the idea of the net infrastructure contributing to the mitigation of urban vulnerability [4,11]. These objectives were also aligned with improving the road condition of the most vulnerable entities in particular, showing that, in some cases, it is possible to reconcile particular with general interest. On the other hand, the risk assessment of the planning alternatives revealed that the closer the alternatives were to the ideal in each objective, the riskier they were. Additionally, the comparison between closeness to ideal and risks showed the existence of turning points in the change of the trend from risk to opportunity that were especially relevant for multi-objective decision-making in the case of conflicting objectives. Regarding the implementation planning, it was possible to find a solution with the best balance between global risk and local opportunity for each objective. However, there was no decentralization solution that performed best for all objectives, which made it necessary to prioritize between objectives and choose accordingly.

Despite the remarkable outcomes, there were still limitations to this study. On one hand, there is still a need for a more systematic approach in the joint analysis of risks of different nature, as is the case. Multi-criteria methods such as AHP [55], Delphi [56], or Bayesian networks [57] can be used to build, based on experts’ or decision-makers’ preferences, a composite implementation risk index that would be of help in the selection of infrastructure planning alternatives. On the other hand, the proposed system of relational contracts may produce legal difficulties requiring specific research to overcome. For example, in Spanish legislation, maintenance and construction activities have different nature and are allocated in separated budget chapters, which requires specific contracts. Breaking down rights and opportunities by provinces in the decentralization framework, and conducting specific research on the interactions between these factors, would also be of use for territorial decision-making. This capacity, which is lacking in the proposed software, could be addressed by programming and incorporating variance-based global sensitivity methods such as Global sensitivity and uncertainty analysis GSUA [49] into the UPSS planning tool code. Finally, this paper studied the relationship between local adaptive capacity and global risks when actions were implemented at a local scale, which in our case was the provincial scale. However, the implementation of actions at this scale is still fragmented, since in provinces there are infrastructures of national, regional, and provincial ownership which are separately operated. In consequence, the framework of relational contracts is directly applicable only to road networks belonging to the same type of ownership, i.e., the networks of the national, regional, or provincial roads. This suggests the need for additional research supporting the development of a system of relational contracts in which the transference of actions between infrastructures of different owners could be regulated in order to achieve duties at the local scale.

## Figures and Tables

**Table 1:** Infrastructure condition variables and planning actions.

Infrastructure/Explanatory Variables:			Possible Action Variables:					
Description	Id	Unit	Type	Treatment Cost (€/m^2^)	SLI (*)	PCI-CS (**)	Treatment/Period (***)	Period Cost
Net Infrastructures:								
Road condition variables								
Road condition: Good	1	m^2^	Preservation	1.02	3	85	4	4
Road condition: Fair	2	m^2^	Maintenance	23.24	10	60	1	23
Road condition: Poor	3	m^2^	Rehabilitation	66.74	25	25	1	67
Road condition: Total	4	m^2^	Construction	496	25	95	1	496

(*) Service life increase, based on Torres-Machí et al. (2017); (**) Pavement condition index condition score, based on Matin et al. (2017) and France-Mensah and O’Brien (2019); (***) Number of treatments required for a 10 year period.

**Table 2:** Actions included in the selected planning alternative for the region of Comunidad Valenciana.

	Initial Road Network Inventory		Actions Planned		Final Road Network Inventory (*)	
	Condition	Quantity (*)	Type	Quantity (*)	Variation	Total
Region: Comunidad Valenciana	Good	101.43	Preservation	90.34	14.34	115.78
	Fair	14.70	Maintenance	12.77	−3.61	11.09
	Poor	2.58	Rehabilitation	2.46	10.56	13.14
			Construction	10.21		
Province 1: Alicante	Good	40.26	Preservation	36.93	3.49	43.75
	Fair	2.83	Maintenance	2.68	0.50	3.33
	Poor	0.32	Rehabilitation	0.30	3.18	3.50
			Construction	3.83		0.00
Province 2: Castellón	Good	20.97	Preservation	16.05	−2.85	18.12
	Fair	0.29	Maintenance	0.25	4.63	4.92
	Poor	0.09	Rehabilitation	0.07	4.89	4.98
			Construction	1.75		0.00
Province 3: Valencia	Good	40.20	Preservation	37.35	13.71	53.90
	Fair	11.58	Maintenance	9.84	−8.73	2.84
	Poor	2.17	Rehabilitation	2.09	2.49	4.67
			Construction	4.62		0.00

(*) Surface in km^2^.

**Table 3:** Results of the global sensitivity analysis of the decentralization model parameters.

Parameter	PDF (*)	Sensitivity Index								
		Economic Cost			Road Condition Improv.			Mitigation of UV		
		1 (**)	2 (**)	3 (**)	1 (**)	2 (**)	3 (**)	1 (**)	2 (**)	3 (**)
Rights:										
Regions (***)	5, 5, 21	8.50%	0.58%	13.67%	5.71%	0.12%	13.67%	10.88%	2.03%	13.67%
Provinces	4, 4, 14	1.34%	87.61%	86.33%	74.64%	99.34%	86.33%	1.73%	96.82%	86.33%
Duties:										
National	1, 1, 3	0.51%	0.01%	0.00%	0.11%	0.00%	0.00%	0.12%	0.00%	0.00%
Region	1, 1, 3	1.80%	0.01%	0.00%	0.47%	0.01%	0.00%	0.01%	0.00%	0.00%
Provinces	1, 2, 6	87.85%	11.79%	0.00%	19.07%	0.53%	0.00%	87.26%	1.15%	0.00%

(*) All parameters’ uncertainties defined by triangular PDF (Min, Peak, Max) points; (**) 1: global risk; 2: bottom opportunity; 3: sum of subnational rights; (***) Summation of the sensitivy index of all spanish regions.

**Table 4:** Guidelines for decentralization alternative 66 for the issuing of relational contracts between central government and the region of Comunidad Valenciana, and between this region and its provinces.

	Rights:	Region	Provinces	Duties:	Region	Provinces
	Range	14%	5%	Range	3%	5%
	Actions	Lb (*)	Ub (*)	Objectives (**)	Lb	Ub
Country and Region: Comunidad Valenciana	Preservation	77.69	101.43	UVM(Net) (−)	−8.77 x 10^3^	−9.31 x 10^3^
	Maintenance	10.98	14.49	RCI(Net) (+)	9.73 x 10^3^	1.03 x 10^4^
	Rehabilitation	2.12	2.56	EC(Net) (−)	5.59 x 10^9^	5.94 x 10^9^
	Construction	8.78	8.25			
Region and Province 1: Alicante	Preservation	35.09	40.26	UVM(Net) (−)	−2.49 x 10^3^	−2.75 x 10^3^
	Maintenance	2.55	2.63	RCI(Net) (+)	3.10 x 10^3^	3.42 x 10^3^
	Rehabilitation	0.29	0.32	EC(Net) (−)	1.98 x 10^9^	2.19 x 10^9^
	Construction	3.64	1.41			
Region and Province 2: Castellón	Preservation	15.25	20.97	UVM(Net) (−)	3.13	3.46
	Maintenance	0.24	0.29	RCI(Net) (+)	3.38 x 10^2^	3.74 x 10^2^
	Rehabilitation	0.06	0.07	EC(Net) (−)	8.79 x 10^8^	9.71 x 10^8^
	Construction	1.67	2.07			
Region and Province 3: Valencia	Preservation	35.48	40.20	UVM(Net) (−)	−6.10 x 10^3^	−6.74 x 10^3^
	Maintenance	9.35	11.58	RCI(Net) (+)	6.09 x 10^3^	6.73 x 10^3^
	Rehabilitation	1.98	2.17	EC(Net) (−)	2.62 x 10^9^	2.89 x 10^9^
	Construction	4.39	4.77			

(*) Surface in km^2^; (**) Negative and positive signs respectively indicate minimization and maximization.
